# An interactive nomogram based on clinical and molecular signatures to predict prognosis in multiple myeloma patients

**DOI:** 10.18632/aging.203294

**Published:** 2021-07-14

**Authors:** Linxin Liu, Jian Qu, Yuxin Dai, Tingting Qi, Xinqi Teng, Guohua Li, Qiang Qu

**Affiliations:** 1 Department of Hematology, Xiangya Hospital, Central South University, Changsha, China; 2 Department of Pharmacy, The Second Xiangya Hospital, Central South University, Institute of Clinical Pharmacy, Central South University, Changsha, China; 3 Department of Biochemistry and Molecular Biology, School of Life Sciences, Central South University, Changsha, China; 4 Department of Pharmacy, Xiangya Hospital, Central South University, Changsha, China; 5 Institute for Rational and Safe Medication Practices, National Clinical Research Center for Geriatric Disorders, Xiangya Hospital, Central South University, Changsha, China

**Keywords:** multiple myeloma, WGCNA, LASSO, overall survival, prognosis

## Abstract

Although novel drugs and treatments have been developed and improved, multiple myeloma (MM) is still recurrent and difficult to cure. In the present study, the magenta module containing 400 hub genes was determined from the training dataset of GSE24080 through weighted gene co-expression network analysis (WGCNA). Then, using the least absolute shrinkage and selection operator (Lasso) analysis, a fifteen-gene signature was firstly selected and the predictive performance for overall survival (OS) was favorable, which was identified by Receiver Operating Characteristic (ROC) curves. The risk score model was constructed based on survival-associated fifteen genes from the Lasso model, which classified MM patients into high-risk and low-risk groups. Areas under the curve (AUC) of ROC curve and log-rank test showed that the high-risk group was correlated to the dismal survival outcome of MM patients, which was also identified in testing dataset of GSE9782. The calibration plot, the AUC value of the ROC curve and Concordance-index showed that the interactive nomogram with risk score could favorably predict the probability of multi-year OS of MM patients. Therefore, it may help clinicians make a precise therapeutic decision based on the easy-to-use tool of the nomogram.

## INTRODUCTION

Multiple myeloma (MM) is an aggressive neoplastic disease characterized by a collection of several diverse cytogenetically distinct plasma cell malignancies with a high degree of heterogeneity, accounting for about 10% of all hematological malignancies [1, 2]. Trisomies and Immunoglobulin heavy chain gene (IgH) translocations are considered primary cytogenetic abnormalities, and other cytogenetic changes, such as gain(1q), del(1p), del(17p), del(13), RAS mutations, and translocations involving MYC, termed secondary cytogenetic abnormalities, also reflect high risk towards MM [3]. Overall survival of MM has significantly improved in the last 15 years, which has moved to the forefront of clinical interest because of the significant advances in medical treatment [4]. MM malignant cells and stromal cells secrete cytokines and growth factors, which explain the biological and clinical manifestations of the disease, including hypercalcemia, renal failure, anemia, bone destruction, and infection [5].

Immunomodulatory drugs such as lenalidomide and pomalidomide, proteasome inhibitors such as bortezomib, autologous stem cell transplantation, and monoclonal antibodies have significantly improved the survival rate of MM patients [6, 7], but recurrence and resistance are still a major problem [8]. The staging and classification of MM have always been the key to MM individualized precision therapy. In clinical practice, even if the International Staging System (ISS) or Durian Salmon Staging (DSS) are similar, the prognosis of MM patients is still different. The differences between these patients may result from a lot of reasons, of which difference in molecular variation may be one of the most important reasons [9]. Thus, a better understanding of the molecular pathogenesis of MM could help us to identify new prognostic and therapeutic targets. With the extensive development, validation, and clinical applications of molecular techniques such as fluorescence *in situ* hybridization and next-generation sequencing, several prognostic and predictive biomarkers have been used to predict progression-free survival, overall survival (OS), and treatment response.

Weighted gene co-expression network analysis (WGCNA), a system biology algorithm, is used to find the correlation patterns among genes across microarray samples, identify modules of high related genes, and relate modules to certain clinical phenotypes [10, 11]. WGCNA is widely used to facilitate the screening or identification of candidate biomarkers or therapeutic targets that are critically associated with clinical traits [12]. The combined analysis of a panel of biomarkers, rather than an individual signature, is the most promising approach that is powerful enough to change clinical management [13–15]. The least absolute shrinkage and selection operator (Lasso), one of the machine learning methods, which uses L1 penalty for penalizing the squared error loss function of the coefficients, is an advanced variable selection algorithm for multi-collinear data or high-dimensional data [16, 17].

In the present study, we combined WGCNA with Lasso regression to simplify the complexity of the network of genes and improve the predictive accuracy of genes to the OS of MM patients. We also constructed a risk score model and an interactive nomogram to predict the prognosis of MM, which may help the clinicians in the treatment of MM in the future. The workflow chart is summarized in Supplementary Figure 1.

## RESULTS

### Construction of WGCNA

**Figure 1:**
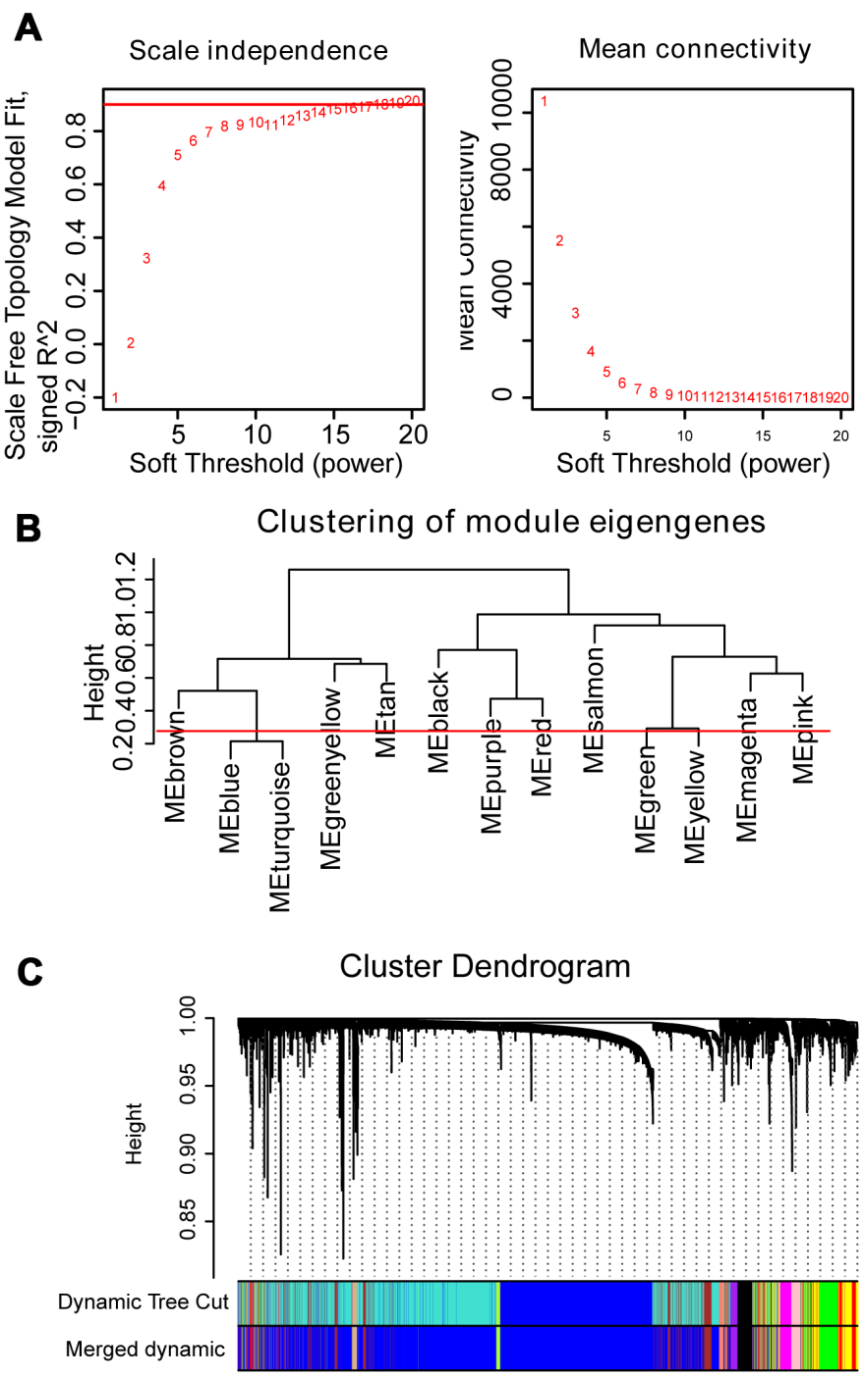
**WGCNA constructs a gene network related to the status of MM.** (**A**) Analysis of the scale-free fit index and mean connectivity for various soft-thresholding powers. The red line indicates the appropriate scale-free topology fit index at 0.9. The best β value was estimated at 0.9. (**B**) Dendrogram of consensus module eigengenes obtained by WGCNA on the consensus correlation. The red line at 0.25 indicates the merge threshold; groups of eigengenes below the threshold represent modules whose expression profiles were merged owing to their similarity. (**C**) Merged modules were identified by the Dynamic Tree Cutting method of WGCNA. Each module is assigned a color as an identifier. According to the correlation between the modules, twelve modules are generated after the merge.

The dendrogram of 554 samples was shown in Supplementary Figure 2, and four outlier samples (GSM592558, GSM592552, GSM592499, and GSM592597) were excluded using the flashclust function of R package “WGCNA”. Heat map of the sample-associated clinical trait was also displayed in the different levels of red color (Supplementary Figure 2). To construct the WGCNA network, we determined the soft threshold power (β=16) to define the adjacency matrix based on the criterion of approximate scale-free topology and mean connectivity (Figure 1A). To figure out the interactions among these thirteen co-expressed modules, the connectivity of eigengenes was analyzed. To construct the co-expressed network and identify the key module, a hierarchical clustering tree for the module was produced using the best-fit β-value (β=16) (Figure 1B). Module blue and turquoise below the cut-off line at 0.25 were merged, resulting in twelve merged modules at last (Figure 1C).

### Identification of most significant modules and eigengenes

**Figure 2:**
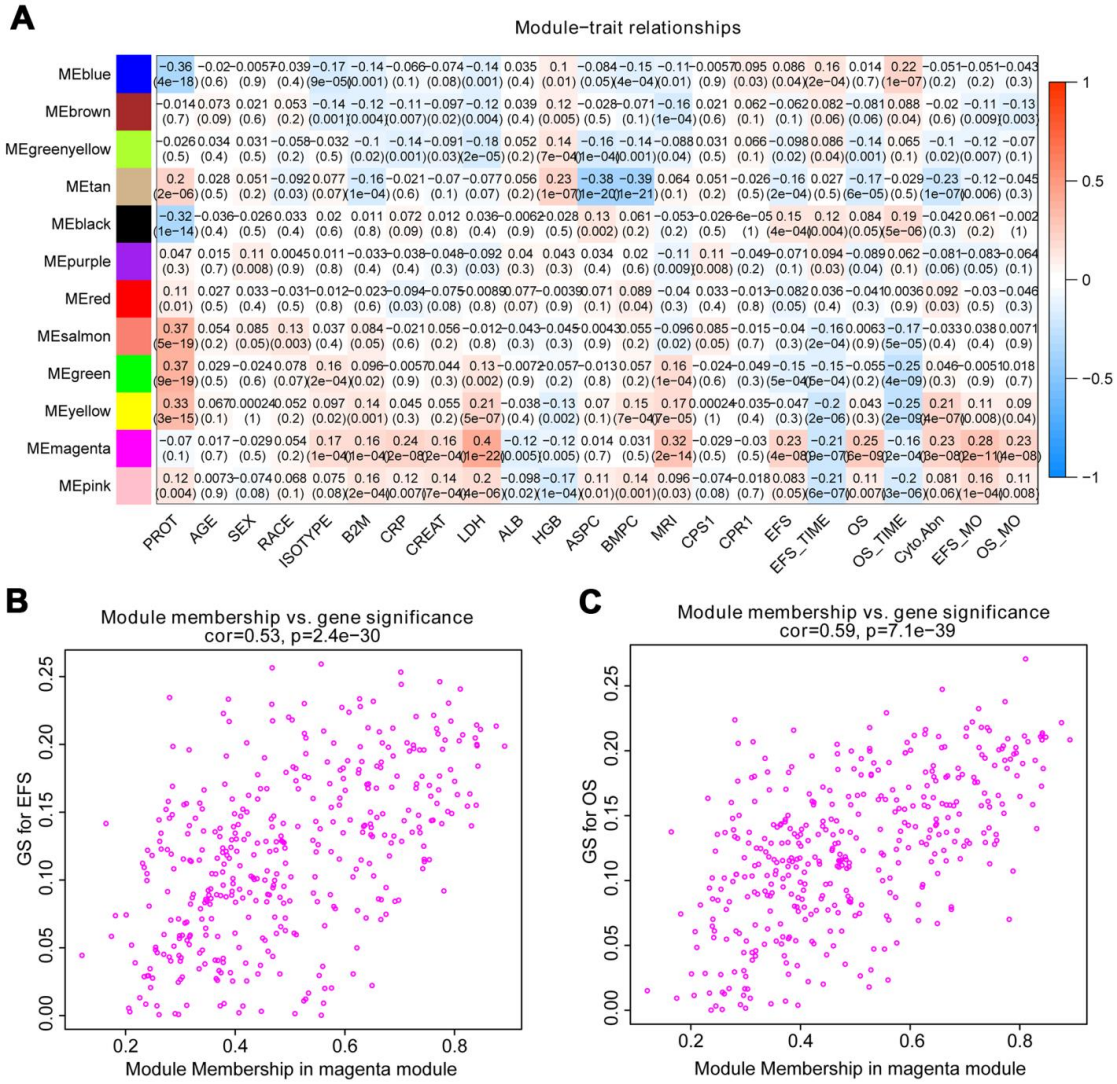
**Identify modules related to the clinical features of MM.** (**A**) Heat map of the correlation between module eigengenes and clinical traits of MM. Red means high adjacency and blue means low adjacency. The correlation coefficient and p-value were listed in the heat map. (**B**, **C**) Scatter plot of module eigengenes related to EFS and OS in the magenta module.

The correlation between module eigengenes and clinical traits of MM was showed in the heat map of Figure 2A. Module magenta was significantly related to event-free survival (EFS) (p=4e-08) and OS (p=6e-09). In addition, the module magenta had the highest GS relating to EFS (p value=2.5e-130) and OS (p value=9e-251) in boxplot (Supplementary Figure 3). Therefore, the magenta module containing 400 eigengenes was used for subsequent analysis. As a result, the relationships between module membership and gene significance of 400 hub eigengenes from the magenta module for OS and EFS were significant, implying that hub genes in the scatter plot also tend to be highly correlated with OS and EFS (Figure 2B, 2C).

### Lasso penalized Cox regression

**Figure 3:**
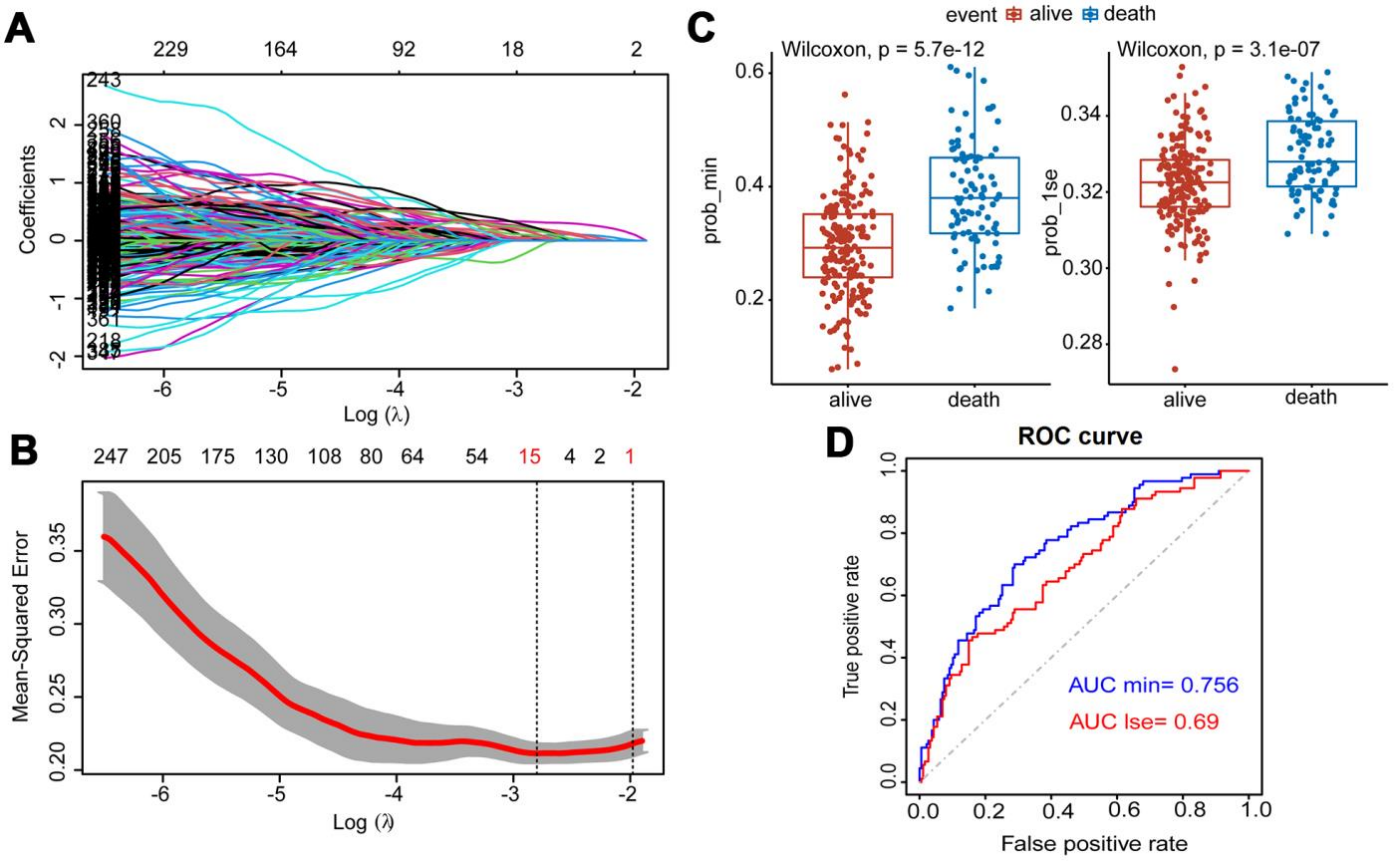
**Construction of Lasso Cox regression model using training dataset of MM.** (**A**) A coefficient profile plot was produced against the log (λ) sequence. (**B**) Tuning parameter (λ) selection in the Lasso model used 10-fold cross-validation via minimum criteria. Dotted vertical lines were drawn at the optimal values by using the minimum criteria and the 1 standard error of the minimum criteria (the 1-SE criteria). (**C**) Survival probabilities (Prob-min and Prob-lse) were predicted by two Lasso models based on two ideal parameters Log λ.lse and Log λ.min. The Wilcoxon test was used to compare the different survival outcomes. (**D**) ROC curves analysis and the values of AUC were used to compare two Lasso models based on Log λlse and Log λmin.

After the Lasso was performed using 400 hub eigengenes using the training dataset, the coefficients were shown in Figure 3A. Genes with non-zero coefficients were considered to have strong prognostic potential in the Lasso penalized regression model. Two tuning parameters (Log λ.min = -2.86 and Log λ.1se = -1.96) were obtained using 10-fold cross-validation via minimum criteria (Figure 3B). Therefore, the fifteen-gene group and one-gene group were obtained based on Log λ.min and Log λ.lse. Wilcoxon test showed that the fifteen-gene group had better survival prediction potential than the one-gene group in MM patients (p<0.05) (Figure 3C). The ROC curves showed that the AUCs of the fifteen-gene group and the one-gene group were 0.756 and 0.69, respectively, indicating that the fifteen-gene group had a more predictive ability of prognosis (Figure 3D).

**Figure 4:**
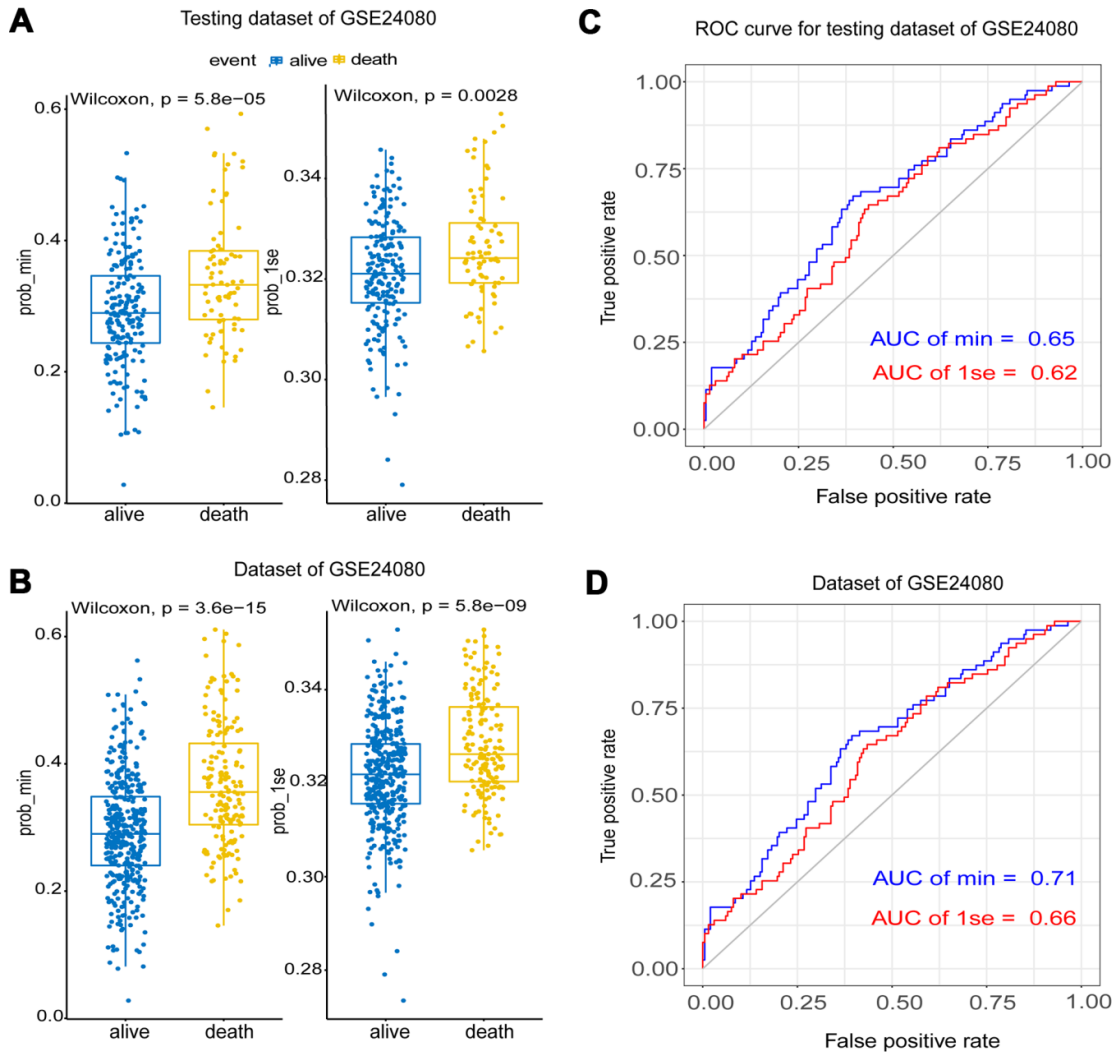
**The validation of the Lasso model using testing and the whole dataset of GSE24080.** (**A**, **B**) Survival probabilities (Prob-min and Prob-lse) were predicted by two Lasso models based on two ideal parameters Log λ.lse and Log λ.min using testing and the whole dataset of GSE24080. The Wilcoxon test was used to compare the different survival outcomes. (**C**, **D**) ROC curves analysis and the values of AUC were used to compare two Lasso models based on Log λ.lse and Log λ.min using testing and the whole dataset of GSE24080.

The testing dataset and whole samples of GSE24080 were used to validate the constructed fifteen-gene and one-gene prognosis models respectively. Wilcoxon test showed that the fifteen-gene prognostic model had better survival prediction potential than the one-gene group in both datasets (p<0.05) (Figure 4A, 4B). The AUCs of the fifteen-gene group and the one-gene group were 0.65 and 0.62 in the testing dataset, respectively, and were 0.71 and 0.66 in the whole sample dataset of GSE24080 (Figure 4C, 4D). As the fifteen-gene model has more accurate prognostic power than the one-gene model, the following studies focused on the fifteen-gene prognostic model.

### Univariate and multivariate Cox regression for fifteen hub genes

To obtain a more accurate and sensitive risk score model, univariate and multivariate Cox regression analyses for fifteen genes were performed. The univariate Cox regression showed that 14 genes could be independent biomarkers for the prediction of OS (p<0.05) (Table 1). Three genes, *AURKA*, *FAM72A*, and *NUF2*, were probably more closely correlated to OS after the multivariate Cox regression performing in the forest plot (p<0.1) (Figure 5A and Table 1). Kaplan-Meier survival analysis also showed that three genes contributed to independent survival prediction (Log-rank p<0.05) (Figure 5B).

**Table 1:** Univariate and multivariate Cox regression analysis for fifteen genes.

**Variable**	**Univariate Cox analysis**			**p**	**Multivariate Cox analysis**			**p**
	**Beta**	**HR**	**95% CI HR**		**Beta**	**HR**	**95% CI HR**	
ABHD3	3.5	34	2.7-440	0.006*	1.205	3.336	0.157-70.765	0.440
ACN9	3.5	32	6.3-160	0.000**	1.461	4.310	0.754-24.640	0.101
ANLN	1.9	6.6	2.1-21	0.001**	-0.097	0.908	0.262-3.145	0.879
AURKA	5	150	31-710	0.000**	2.442	11.43	1.412-93.551	0.023*
CTNNAL1	0.79	2.2	0.34-14	0.410	-0.931	0.394	0.059-2.628	0.336
FAM210A	6.5	660	24-18000	0.000**	-0.292	0.747	0.017-32.382	0.880
FAM72A	4.1	62	19-200	0.000**	2.301	9.983	1.282-77.727	0.028*
HAUS3	4.9	140	5.6-3400	0.003*	1.835	6.264	0.204-191.915	0.293
KNSTRN	7.3	150	54-39000	0.000**	2.991	19.99	0.433-915.781	0.126
LINC00998	5.3	200	16-2600	0.000**	1.084	2.958	0.159-54.955	0.467
LOC81691	1.3	3.7	1.9-7.5	0.000**	0.387	1.472	0.666-3.253	0.339
NUF2	2.3	10	4.1-26	0.000**	-1.105	0.331	0.099-1.108	0.073#
SMCHD1	5.1	160	10-2400	0.000**	2.310	10.08	0.421-240.952	0.154
TIMM21	6.6	710	41-12000	0.000**	1.788	5.980	0.154-232.174	0.338
ZNF92	6	380	27-5600	0.000**	1.026	2.791	0.148-52.590	0.493

#, p<0.1, *, p<0.05, **, p<0.001.

**Figure 5:**
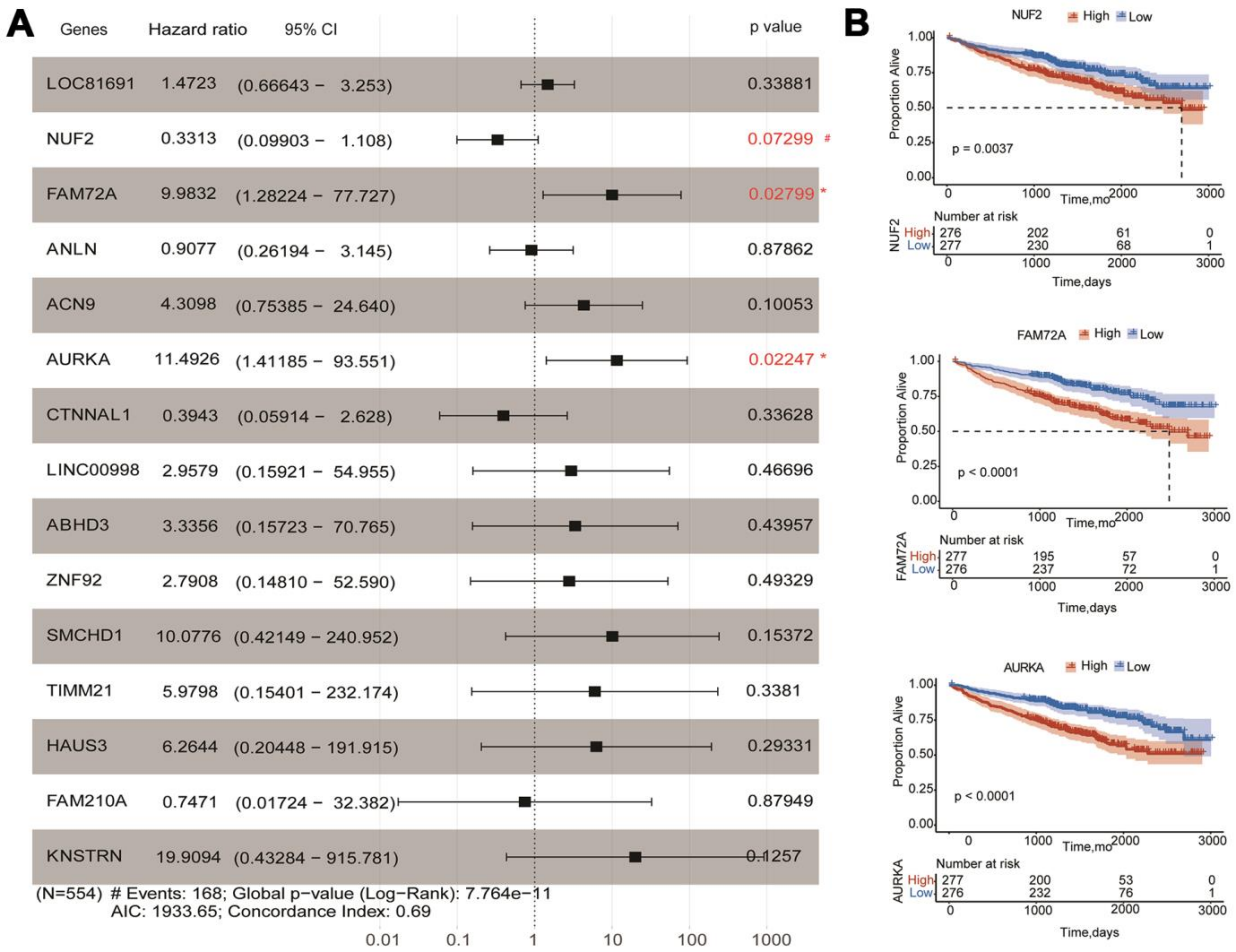
**Effects of hub genes on survival outcome of MM patients of GSE24080.** (**A**) Forest plot of multivariate Cox regression analysis for fifteen genes. FAM72A, AURKA, and NUF2 were significantly associated with survival in multiple myeloma patients (p<0.1). (**B**) Kaplan-Meier analysis of FAM72A, AURKA and NUF2 to predict patient survival. MM patients with the high and low expression levels of three genes were divided into two groups according to the cut-off value of medium expression levels. p-value of the Log-rank test less than 0.05 was considered as a statistical difference.

### Establishment of a fifteen-gene risk score model

**Figure 6:**
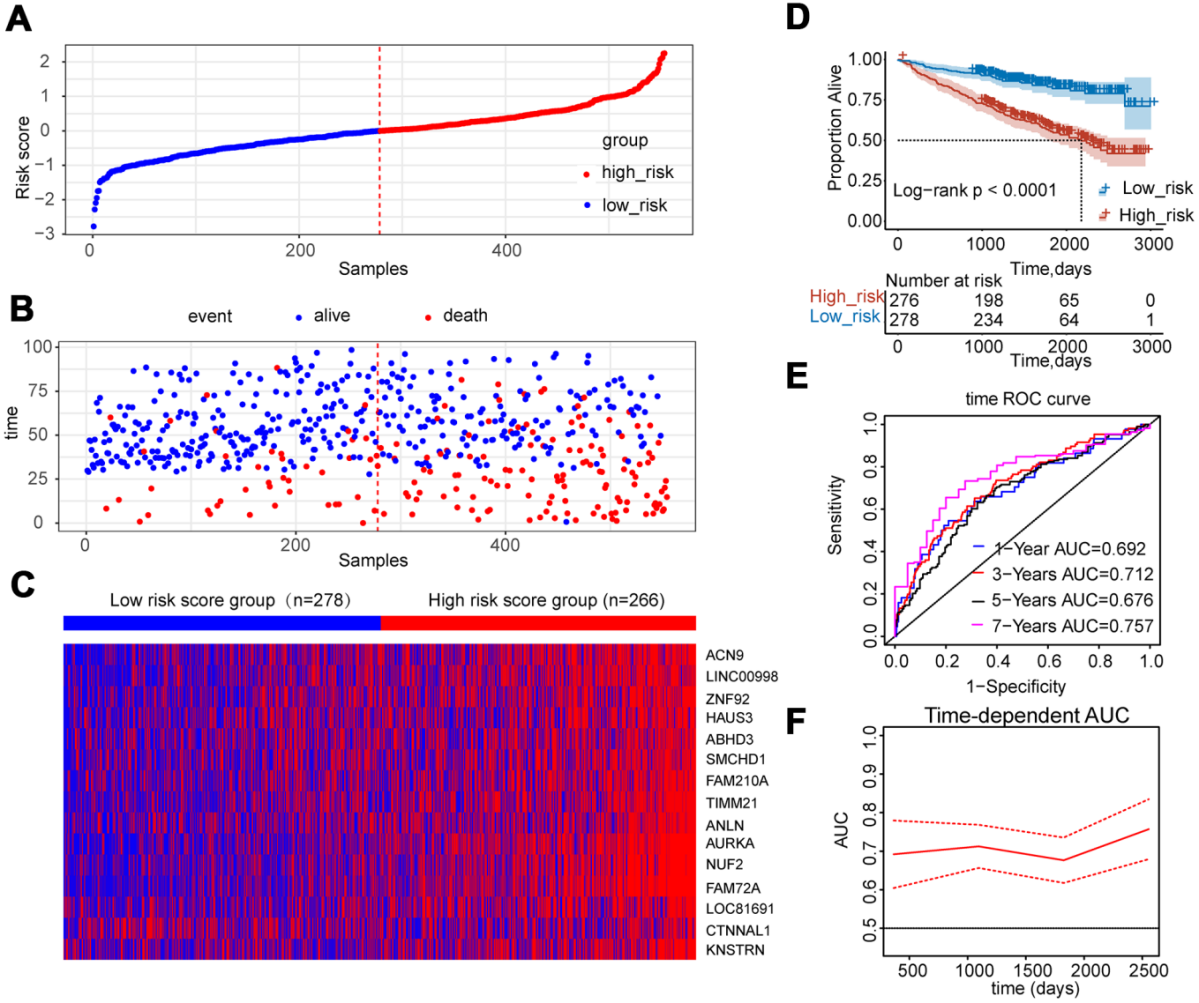
**Construction of risk score model using the fifteen-gene expression profile in MM patients of GSE24080.** (**A**) Fifteen-gene risk score distribution of MM patients based on risk score levels. The best cut-off value was used to divide the patients into two groups. (**B**) The survival status of all patients was distributed and classified by risk score group. (**C**) The expression profiles of the fifteen genes in high-risk and low-risk groups. (**D**) Kaplan-Meier analysis of the fifteen-gene risk score model. (**E**) Time-dependent ROC curves for the fifteen-gene risk score model to predict overall survival of MM patients. AUCs for 1 to 7-year survival were shown in the figure. (**F**) Time-dependent AUCs for the risk assessment model.

A fifteen-gene risk score model was built by multivariate Cox hazard regression analysis. The Concordance-index value was 0.69 and the global log-rank p-value was 7.764e-11 (p<0.05) in the forest plot (Figure 5A), indicating that the risk score model containing fifteen hub genes could be used to predict OS. MM patients with a risk score ≥ 0 were classified into the high-risk group, whereas MM patients with a risk score<0 were divided into the low-risk group. Compared to the low-risk group, the distribution plot of survival status showed that the samples of death events were enriched in the high-risk group (Figure 6A, 6B). All genes were overexpressed in the high-risk group in the heat map (Figure 6C). Consistently, the Kaplan-Meier curve showed that the high-risk group had lower survival possibility, compared to the low-risk group (Log-rank p < 0.0001) (Figure 6D). Time-dependent ROC curves further verified and depicted the excellent prediction performance of the fifteen-gene risk score model with the AUC of 1-year was 0.692, 3-year AUC =0.712, 5-year AUC =0.676, 7-year AUC =0.757 (Figure 6E). The mean of time-dependent AUC was over 0.7 during the different time, which imply that the risk score model of fifteen genes may contribute to accurate survival prediction (Figure 6F). Six hub genes were selected from the testing dataset of GSE9782 and used to construct a risk score model in Supplementary Table 1. The similar results showed that this risk score model was also beneficial for the prediction of survival based on log-rank test p<0.0001 and AUC value was 0.638.

### Establishment and validation of the nomogram

**Figure 7:**
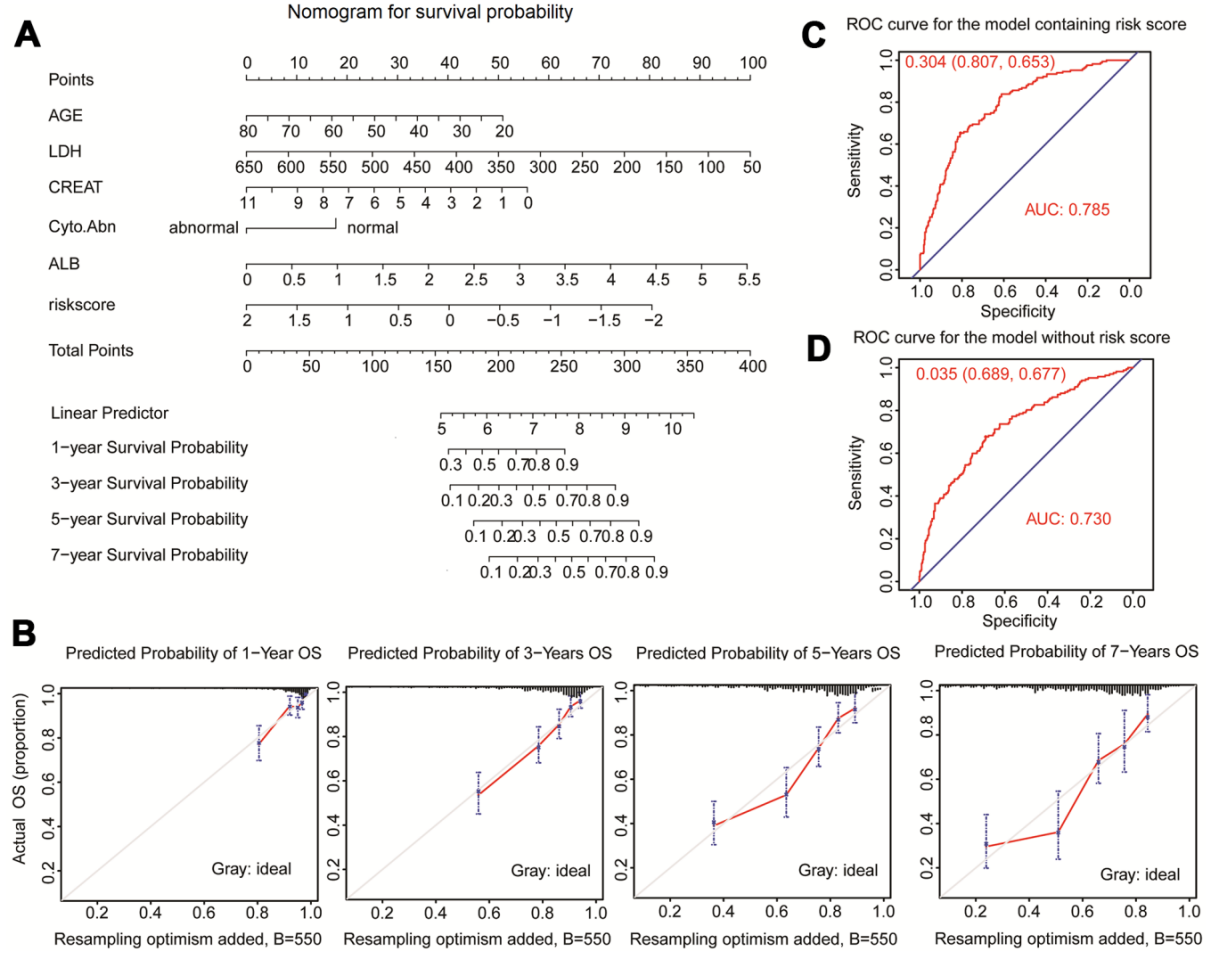
**Nomogram predicting 1-year, 3-year, 5-year, and 7-year overall survival of multiple myeloma patients.** (**A**) The nomogram consists of the fifteen-gene risk score and five clinical risk indicators. Add the points from these 6 variables together and find the location of the Total Points. The Total Points projected on the bottom scales indicate the probability of 1-year, 3-year, 5-year, and 7-year overall survival. (**B**) The calibration curve for predicting 1-year, 3-year, 5-year, and 7-year overall survival. (**C**) ROC curves for the nomogram model with risk score to predict patient survival. (**D**) ROC curves for the nomogram model without risk score to predict patient survival.

**Figure 8:**
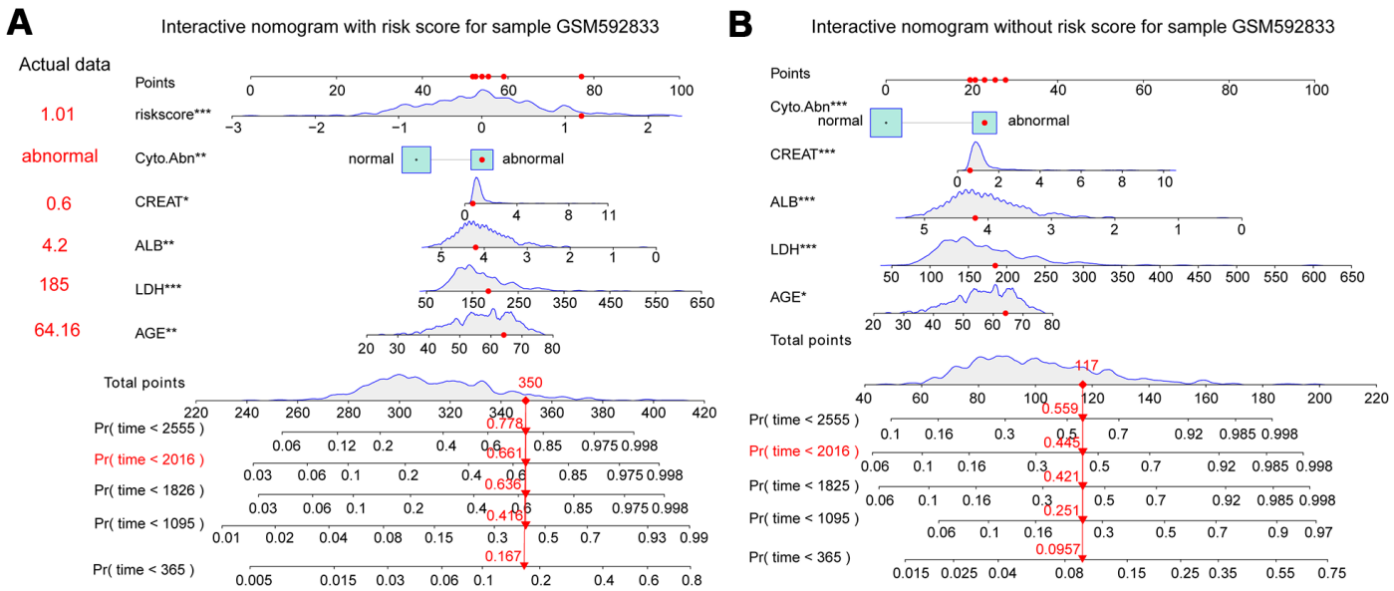
**Interactive nomogram predicts the survival probability of overall survival for the GSM592833 sample.** The patient died on the following day of 2016. **(p<0.01), ***(p<0.001) indicates a significant correlation. (**A**) Nomogram containing five clinical traits and fifteen-gene risk score predicts 365-day, 1095-day, 1825-day, 2016-day, and 2555-day overall survival probability. (**B**) A nomogram containing five clinical traits without a fifteen-gene risk score predicts overall survival probability.

Clinical traits and the fifteen-gene risk score were also analyzed by univariate and multivariate Cox regression to identify the survival-related variable. The risk score and clinical signatures including AGE, CREAT, LDH, ALB, Cyto_Abn were found to be significantly related to OS (p<0.05) (Supplementary Table 2). Since five clinical traits and risk scores were vital variables for the prediction of OS of MM, we rebuilt the multivariate Cox regression model and visualized it by nomogram. Total points were summated by adding each point of AGE, CREAT, LDH, ALB, Cyto_Abn, and the fifteen-gene risk score (Figure 7A). The calibration curves of 1 to 7-year survival demonstrated favorable prediction performance of nomogram (Figure 7B). To illustrate the significant influence of the fifteen-gene risk score in the nomogram, the ROC curve further verified that AUC for the model containing the risk score was 0.785, higher than 0.730 without risk score (Figure 7C, 7D). Meanwhile, the C-index of the model with the risk score was 0.754, higher than 0.722 of the C-index without the risk score (Table 2). The patient sample of GSM592833 was randomly selected and used to illustrate the nomogram (Figure 8A, 8B). It should be noted that the patient died on the following day of 2016. The death probability of this sample was 0.661 predicted by nomogram with a risk score, but only 0.445 predicted by the nomogram without risk score. These results indicate that the nomogram with risk score has better predictive performance thus is more reliable. According to the equation of nomogram, after inputting the values of clinical traits and risk score, the prediction of OS was estimated via nomogram tool in Microsoft Excel (Supplementary Table 3).

**Table 2:** Nomogram model parameters.

**Variable**	**Nomogram with risk score**			**p**	**Nomogram without riskscore**			**p**
	**Beta**	**OR**	**95%CI**		**Beta**	**OR**	**95%CI**	
AGE	0.024	1.024	1.007-1.042	0.005*	0.021	1.021	1.004-1.039	0.013543*
LDH	0.004	1.004	1.002-1.006	0.000**	0.005	1.005	1.004-1.007	6.08e-09**
ALB	-0.334	0.716	0.572-0.897	0.004*	-0.450	0.637	0.512-0.793	5.32e-05**
CREAT	0.101	1.106	1.013-1.208	0.025*	0.146	1.157	1.062-1.261	0.000895**
Cyto-Abn	-0.511	0.600	0.436-0.826	0.001*	-0.699	0.497	0.365-0.677	9.39e-06**
risk score	0.647	1.910	1.501-2.430	0.000000145**	NA	NA	NA	NA
C-index	0.754±0.019				0.722±0.02			
Likelihood ratio test	122.6, p<0.001				94.85, p<2e-16			
Wald test	138.6, p=<0.001				111.5, p=<2e-16			
Score (Log-rank test)	150, p=<0.001				119.3, p=<2e-16			

*, p<0.05, **, p<0.001.

## DISCUSSION

Although several new drugs have been introduced into clinical practice, MM is still incurable with dismal clinical outcomes in MM patients [18, 19]. Molecular risk stratification basing on hub gene expression of MM has opened an avenue for clinicians to conduct personalized medicine [20, 21]. Therefore, it is critical to developing novel molecular biomarkers that are closely related to the clinicopathological characteristics and clinical survival outcomes of MM patients.

To reveal the relationship between clinical information and gene expression matrix, many bioinformatics methods are developed. A prognosis-associated long noncoding RNA-mRNA network for multiple myeloma was constructed by WGCNA based on the microarray of MM (GSE24080) [22]. In the present study, we firstly comprehensively investigate the influence of genes on the prognosis of MM patients via WGCNA. The key module magenta was screened out according to the p-value and Pearson’s coefficients in the module-trait heat map. The bar plots of gene significance also showed that the module magenta was highly correlated to EFS and OS (p<0.05). Dot plot of gene significance and module membership revealed that 400 hub genes within the magenta module were significant.

To further screen hub genes involved in the prognosis of MM, Lasso was introduced to improve the prediction accuracy by forcing the sum of the absolute value of the regression coefficients to be less than a fixed value. We incorporated the 400 hub genes into the Lasso model, fifteen hub genes with non-zero coefficients in the model were identified based on log λ.min. The ROC curves indicated that the fifteen-gene model showed better performance of prediction ability compared to the one-gene model based on log λ.lse. Multivariate Cox regression analyzed the predicted attribution of individual genes within the fifteen-gene model and three genes (*AURKA*, *NUF2*, and *FAM72A*) were found to be significantly correlated to survival outcome (p<0.1) the same as independent indicators using the K-M curve (Log-rank p<0.01). Considering the interaction and influence among hub genes, we constructed the risk score model to weigh the efficacy of fifteen genes on OS. The low-risk score group had a better survival outcome compared to the high-risk score group. The classifier based on risk score was well performed to distinguish the status of OS by time ROC curve. The risk score model containing six hub genes was constructed using the testing dataset of GSE9782, which also showed favorable prediction ability.

Univariate and multivariate Cox regression analyses were also used to identify favorable clinical risk characteristics, at last, AGE, LDH, CREAT, Cyo_Abn, and ALB were selected due to the high correlation to prognosis (p<0.05). Compared to the previously published nomogram, it is worth mentioning that we considered gene risk score as a continuous variable which was more accurate to predict prognosis. This research not only considered one aspect of gene expression, but the clinical characters and risk gene combination, eventually integrated into the nomogram, intuitively reflect effects of each part on the prognosis of patients with MM, and ultimately to the scores, for 1 to 7 years survival rate of prediction. Thus, an interactive nomogram was established, taking into account a variety of survival-related five clinical traits and the fifteen-gene risk score. ROC curve analysis and C-index showed that the nomogram with risk score had higher reliability than the model without a risk score. Meanwhile, the nomogram model without the fifteen-gene risk score was also validated in the same sample of GSM592833, but with less accuracy.

Compared to the R-ISS system, LDH and cyto_abnormaty were also considered as indicators in the nomogram. The continuous values of LDH were transferred to points of the nomogram, which was more accurate for the prediction of the survival of each patient. Another prognostic model containing the ISS system, the expressions of two miRNA (let-7b and miR-18a) in serum and cytogenetics, could improve the identification of patients with newly diagnosed MM with poor outcomes [23]. The AUC was near 0.73 lower than the AUC value of 0.785 in our nomogram model. Other models using Gaussian process regression and random forests model also illustrated that the AUC value was near 0.70 lower than the AUC value of 0.785 in our nomogram model [24].

The molecular signature of these hub genes has been demonstrated to be associated with the proliferation or invasion of several human cancers. *AURKA* encodes a serine/threonine kinase located in the centrosomes and plays a vital role in the distribution of chromosomes to two daughter cells in mitosis by participating in the replication, separation, and maturation of centrosomes [25, 26]. Studies have shown that overexpression of *AURKA* leads to chromosome instability and promotes malignant transformation of cells [27]. AURKA phosphorylates p53 serine 315 residues, promoting MDM2-mediated degradation, while AURKA silencing reverses this process. Besides, reduced AURKA levels led to greater stability, while increased AURKA expression undermined the response to cisplatin-induced apoptosis [28, 29]. AURKA protected ovarian cancer cells from chemotherapeutic drug-induced apoptosis by activating the Akt pathway in a p53-dependent manner [30]. There is increasing evidence that AURKA is expressed to varying degrees in newly diagnosed and recurrent MM patients and MM cell lines [30, 31]. AURKA has been reported to be associated with myeloma resistance and early disease recurrence [32]. AURKA may disrupt the DNA damage repair response by regulating DNA repair proteins such as CHK1/2 [33]. Inhibition of AURKA expression in MM cells induced apoptosis and death [31, 34, 35]. Some small molecule inhibitors against AURKA are currently being studied in clinical trials in MM or other cancer patients [36, 37]. In the present study, we also showed that AURKA could be an independent indicator for the prediction of the survival outcomes of MM patients.

NUF2, also named CDCA1 as a part of a protein complex associated with the centromeres, is essential for normal centromere microtubule attachment [38] and chromosome instability in tumor cells [39]. Abnormal expression of NUF2 leads to mitotic dysregulation [40, 41]. The dysexpression of NUF2 is closely related to the development of tumors and it can be used as a biomarker for poor prognosis [42]. Small interfering RNA inhibited NUF2 expression in pancreatic cancer, glioma, and liver cancer and reduced the growth of tumors [43, 44]. Besides, the sub-G1 proportion in the cell cycle was significantly increased [44, 45]. The prognostic impact of NUF2 correlates well with the dismal prognosis of MM [46]. After the multivariate Cox regression analysis was performed, it appears that lower expression of NUF2 was associated with worse outcomes in the forest plot, but the K-M curve showed the improved outcome for low expressions, which was different may due to the differences in statistical methods and the influence by gene-gene interaction. It implied that our interactive nomogram with risk scores could more favorably predict the probability of multi-year OS of MM patients than single gene expression.

FAM72A (family with sequence similarity 72 member A), known as p17 or LMPIP, is composed of 149 amino acids as a kind of neuron protein [47]. Under physiological conditions, FAM72A is expressed at low levels; but the overexpression can cause neuronal cell death. This protein has been shown to have high clinical relevance for survival/death outcomes in cancer patients, as it may be associated with tumorigenic effects in non-neuronal tissue including breast, colorectal, and lung cancer [48–50]. Preliminary data show that FAM72A acts downstream of PKC as a uracil DNA glycosylase-2 (UNG2) binding protein and mediates tumorigenic action [47]. Cell cycle analysis showed that FAM72A drove cells into the G0/G1 phase, which may explain the physiological low expression level of FAM72A in proliferating cells and its role in neurons [49]. Tumor cells may attempt to compensate for the inhibition of p53 and the interference of base excision repair (BER) mechanism, so it may be characterized by the increased expression level of FAM72A protein [48]. This was consistent with previous cell cycle analysis by Wang et al. which showed that FAM72A shortened G1/S transition in nasopharyngeal carcinoma [51].

Other hub genes were also reported in various types of cancers as independent indicators for survival prediction. Rare homozygotes in the *ANLN* (rs12535394) SNP pair are prognostic of favorable breast cancer survival [52]. High levels of ANLN contributed to the poor prognosis of anthracycline-based chemotherapy in breast cancer patients [53, 54]. A three protein-coding genes prognostic model including *SMCHD1* predicts overall survival in bladder cancer patients [55]. Univariate and multivariate survival analysis showed that high expression of *HAUS3* was an independent prognostic factor for the dismal outcome of overall survival of HCC patients [55]. High expression of HAUS3 is also associated with poor prognosis and HCC progression [56]. KNSTRN is significantly associated with dismal survival status in endometrial cancer [57].

In summary, this study used a variety of analytical methods to establish a scoring system as a prognostic indicator for MM patients. The prognostic model was an independent classifier for MM. Moreover, several hub genes in the model could be utilized as an effective drug target for MM treatment.

## MATERIALS AND METHODS

### Data collection and preprocessing

The microarray expression data of GSE24080 as the training dataset and GSE9782 as the testing dataset of MM patients were obtained from the Gene Expression Omnibus database (http://www.ncbi.nlm.nih.gov/geo). According to the annotation information, the probes are converted into gene symbols on the array platform, and the gene expression is calculated by R package “limma”. All samples with complete follow-up clinical information are listed in Supplementary Figure 4.

### Construction of WGCNA

The details of the WGCNA algorithm were conducted by the R package “WGCNA” and were described in our previous publications [58–60]. Firstly, we used the sample dendrogram clustering method to remove the obvious outlier samples with too many missing entries for outlier detection and showed in a clinical trait heat map. Secondly, based on the criteria of approximate scale-free topology and mean connectivity, the soft-threshold power was calculated by the pickSoftThreshold function of WGCNA. Thirdly, we calculated adjacencies using best-fit soft-threshold power, transformed the adjacency into a topological overlap matrix (TOM), and calculated the corresponding dissimilarity TOM (dissTOM). Finally, after hierarchical clustering analysis based on the dissTOM, modules were generated by the dynamic tree cut method for module merging (the cut-off line at 0.25).

### Construction of module-trait relationships and identification of hub modules associated with clinical traits

Pearson’s correlation coefficients and p values between modules and MM clinical traits were calculated and visualized in a module-trait heat map. The module membership represents the correlation between the clinical traits and MM’s clinical status. The average gene significance (GS) in each module was displayed in a bar graph, which also reflected the relationship between the module and MM clinical status. The module with the highest module significance and module membership was considered as hub clinical module. To further identify hub eigengenes within key modules, a scatter plot of GS related to module membership for clinical traits was visualized. The p values and Pearson’s coefficients were also calculated.

### Lasso penalized regression analysis

The expression matrix of eigengenes within the key module was divided into the training and testing datasets of GSE24080 by the R package of “caret”. Lasso penalized regression was used to select the most powerful predictive eigengenes from the training dataset. By performing L1 norm regularization through the R package “glmnet”, different coefficients corresponding to different λ values were obtained. The “cv.glmnet” function was used to select the optimal λ value by ten-fold cross-validation. Gene screening using Log λ.min or Log λ.1se was conducted by “coef” function. Comparison analysis of survival probability of the two ideal models was conducted by the Wilcoxon test and ROC curves through the R package of “pROC” using the training dataset, testing dataset, and the whole expression matrix of GSE24080.

### Univariate and multivariate Cox regression analysis

To further identify the influence of hub genes and clinical traits on the survival outcome of MM, univariate and multivariate Cox regression analyses were conducted using R packages “survival” and “survimer”. A p-value less than 0.05 was considered statistical significance. Kaplan-Meier (K-M) curve and Log-rank test were also conducted to compare survival outcomes between high-expression and low-expression groups by R packages “survival” and “survimer”. Log-rank p<0.05 was considered statistical significance.

### Establishment of risk score model based on fifteen-gene signature

The risk score for MM patients was calculated and evaluated by Concordance-index (C-index) based on the Cox proportional-hazards model [21, 58]. Then all patients were divided into a high-risk group and a low-risk group according to the cut-off value of the risk score. The risk score distribution, survival status, and expression pattern of the fifteen hub genes were displayed in the dot plot and heat map. Kaplan-Meier analysis was used to compare survival outcomes between the high-risk group and low-risk group. Log-rank p<0.05 was considered as statistical significance. Time-dependent ROC analysis was performed and the time-dependent area under the curve (AUC) was calculated by the R package “timeROC”. The testing dataset of GSE9782 was used to testify the performance of the risk score model following the above process.

### Nomogram

Five indicators including AGE, lactate dehydrogenase (LDH), creatinine (CREAT), cytogenetic abnormalities (Cyto_Abn), and the fifteen-gene risk score were enrolled to establish the nomogram by the R package “rms”. The interactive nomogram was calculated by the function of R package “glm” and showed using “regplot”. Each indicator had its point, and the total points were calculated by adding all the points together. The total points of these indicators and survival probability were calculated by the R package “nomogramEx”. C-index was measured to evaluate the discrimination performance of nomogram with or without risk score by R package “Hmisc”. The calibration curve and ROC curve were also plotted to evaluate the prediction efficacy of the nomogram with or without risk score. The nomogram was validated using a randomly selected sample of GSM592833 to predict the 1-year, 3-year, 5-year, and 7-year OS. A predictive tool of a nomogram based on Microsoft Excel was developed for convenient clinical use. After the input of clinical information and the risk score of MM patients, the survival probability is output (Supplementary Table 4).

### Availability of data and materials

The datasets used and/or analyzed during the present study are available from the corresponding author on reasonable request.

## Supplementary Material

Supplementary Figures

Supplementary Table 1

Supplementary Table 2

Supplementary Table 3

Supplementary Table 4
