# Supplementary material for: An interactive nomogram based on clinical and molecular signatures to predict prognosis in multiple myeloma patients
Source: Aging (Albany NY). 2021 Jul 14;13(14):18442–63. doi: 10.18632/aging.203294 (PMC8351694; doi:10.18632/aging.203294)
Supplement: Supplementary Figures [file aging-13-203294-s001.pdf]

SUPPLEMENTARY FIGURES

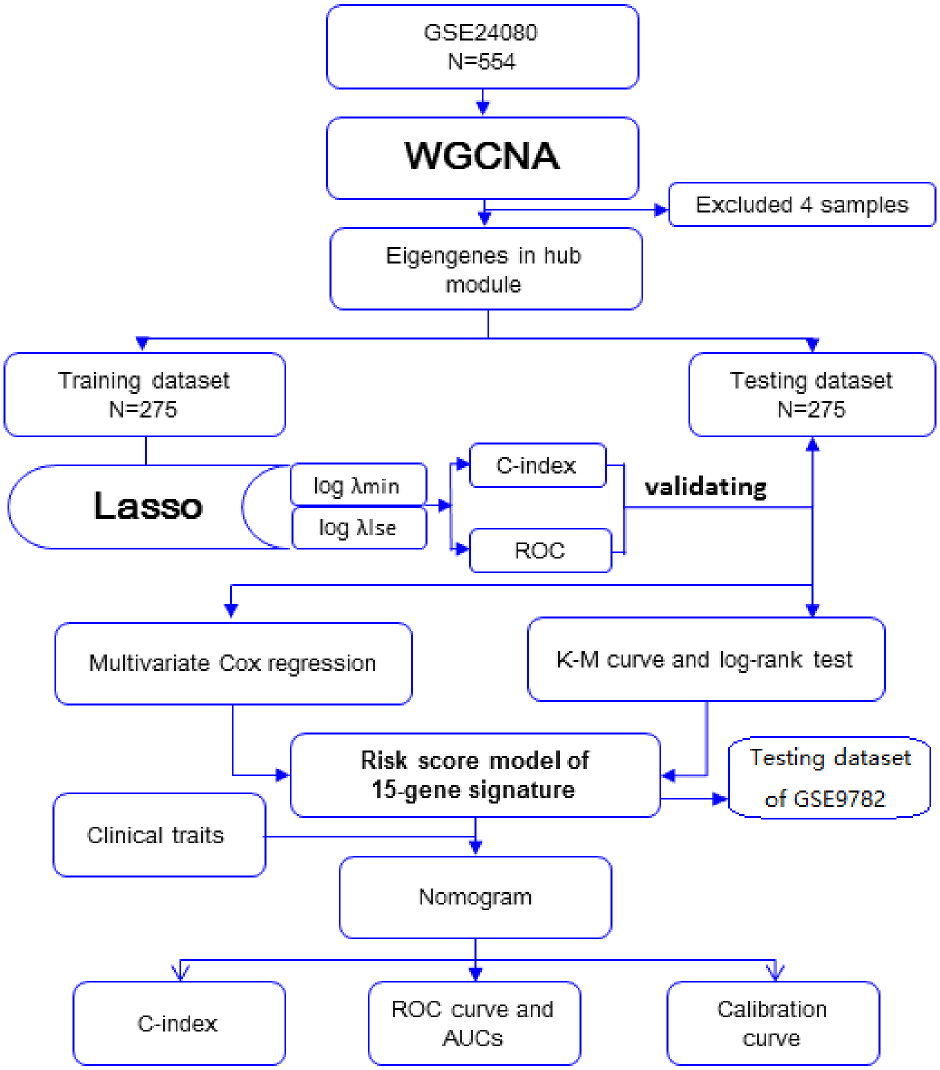

Supplementary Figure 1. The workflow chart of this study.

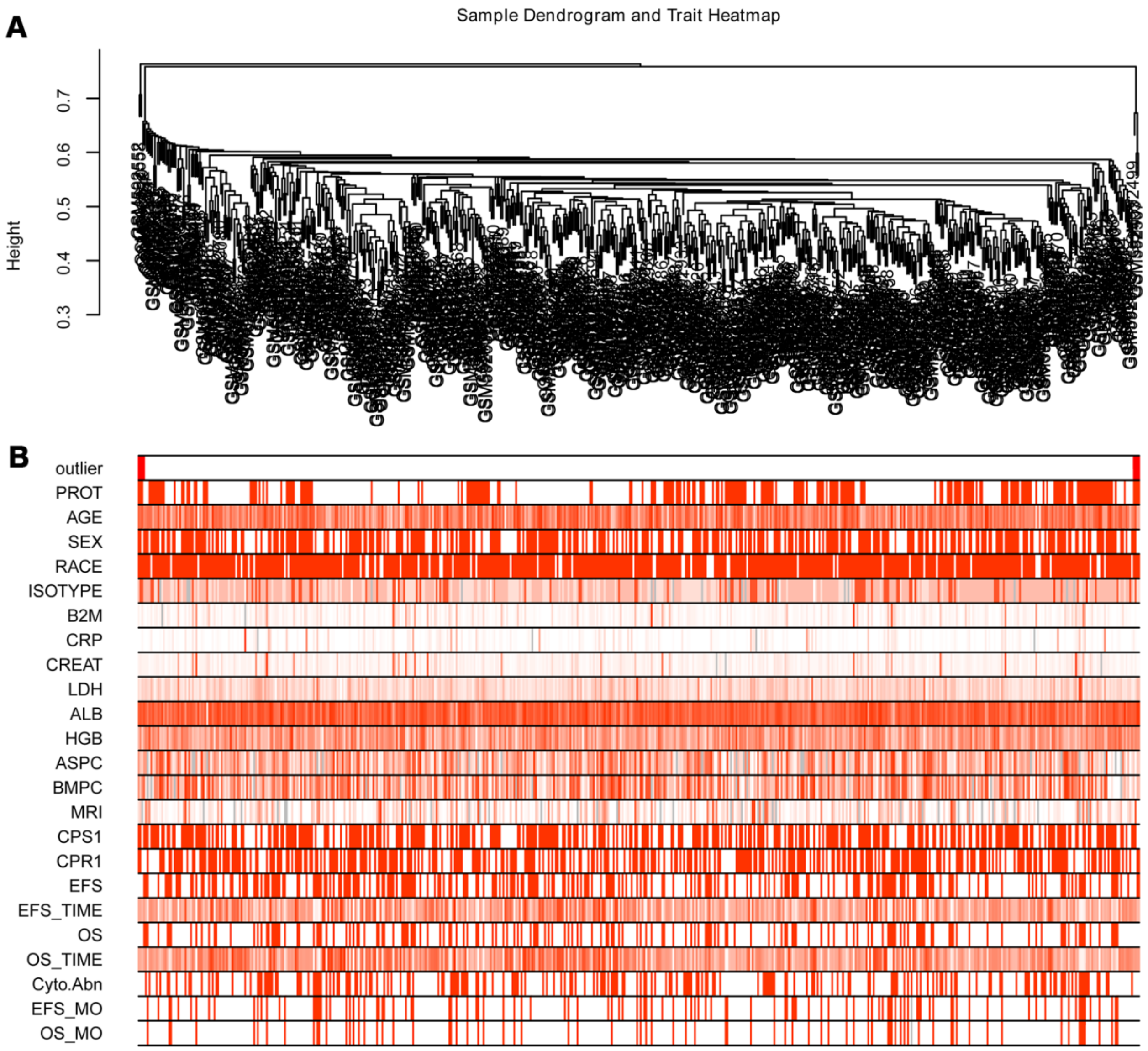

Supplementary Figure 2. WGCNA construction. (**A**) Clustering dendrogram of 554 samples of MM. Four outlier samples (GSM592558, GSM592552, GSM592499 and GSM592597) were excluded using the flashclust function of R package "WGCNA". (**B**) Heat map showed the clinical datasets of MM patients.

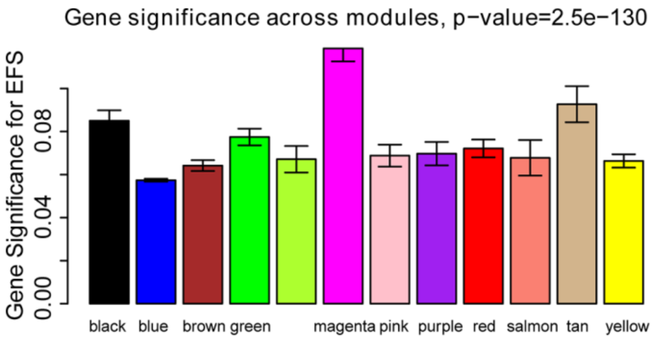

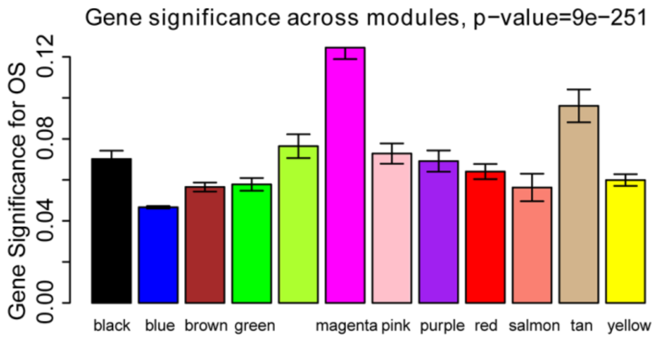

Supplementary Figure 3. Distribution of average gene significance and errors in the modules associated with EFS and OS of MM.

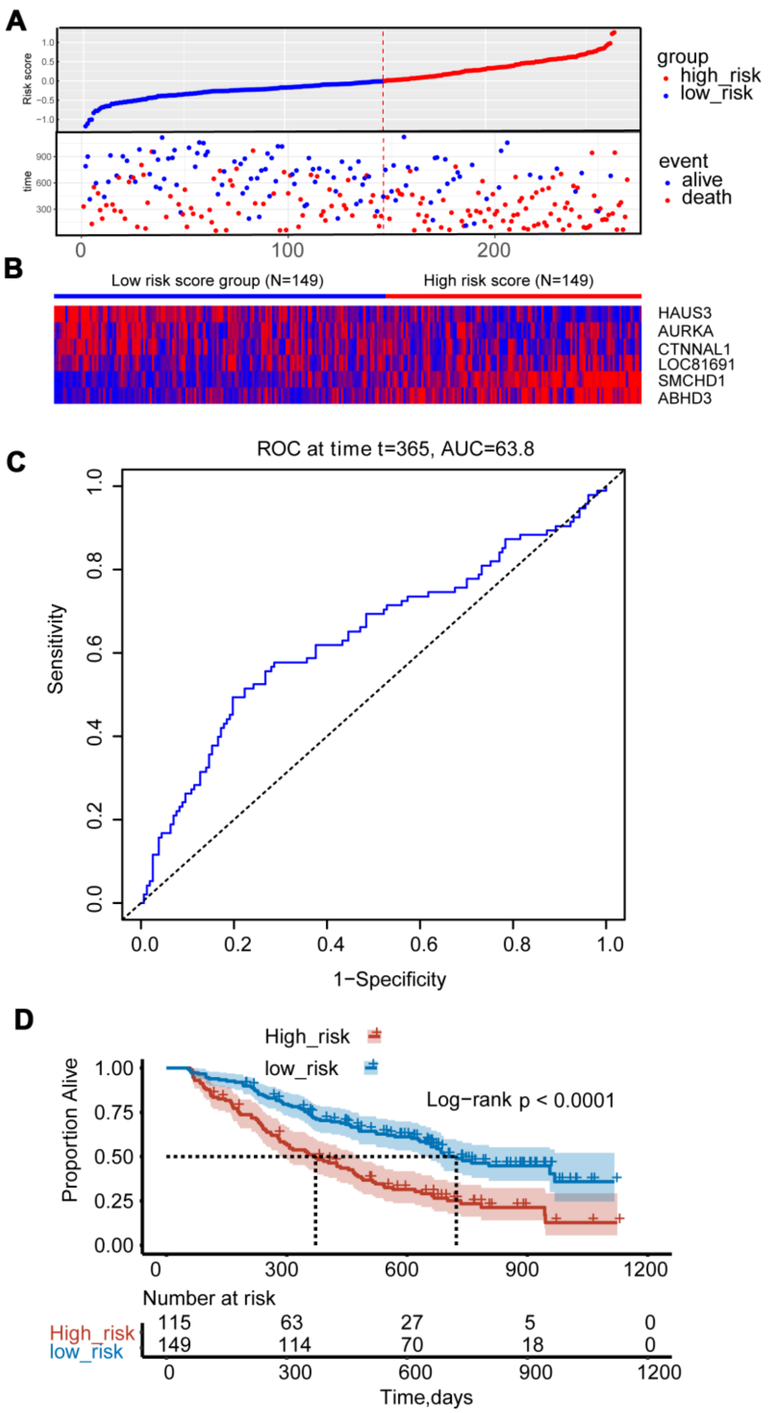

Supplementary Figure 4. Risk score model for GSE9782. (**A**) Construction of risk score model using the fifteen-gene expression profile in MM patients of GSE9782. (**A**) The selected six-gene risk score distribution of MM patients based on risk score levels. The best cut-off value was used to divide the patients into two groups. The survival status of all patients was distributed and classified by risk score group. (**B**) The expression profiles of six genes in high-risk and low-risk groups. (**C**) ROC curve for the fifteen-gene risk score model to predict overall survival of MM patients. AUC was 0.638. (**D**) Kaplan-Meier analysis of the six-gene risk score model.
