# Supplementary material for: An interactive nomogram based on clinical and molecular signatures to predict prognosis in multiple myeloma patients
Source: Aging (Albany NY). 2021 Jul 14;13(14):18442–63. doi: 10.18632/aging.203294 (PMC8351694; doi:10.18632/aging.203294)
Supplement: Supplementary Table 3 [file aging-13-203294-s004.pdf]

SUPPLEMENTARY TABLE

Supplementary Table 3. Summary of clinical characteristics of GSE24080.

Items		Input data		
AGE	1 year - 80 years	**64.16**	**years**	
CREAT	Creatinine	**0.6**	**mg/dl**	
LDH	Lactate dehydrogenase	**185**	**U/l**	
ALB	Albumin	**4.2**	**g/dl**	
Cyto_Abn	An indicator of the detection of cytogenetic abnormalities	**1**	**1=abnormal; 0=no detected or absent**	
Riskscore	Risk score based on genes	**1.01**	**1=high risk; 0=low risk**	
**Results:**	1-year Survival Prob			**0.858645467**
	3-years Survival Prob			**0.61493303**
	5-years Survival Prob			**0.408277594**
	7-years Survival Prob			**0.271462399**

Nomogram predicts overall survival of MM patients.
