# Supplementary material for: An interactive nomogram based on clinical and molecular signatures to predict prognosis in multiple myeloma patients
Source: Aging (Albany NY). 2021 Jul 14;13(14):18442–63. doi: 10.18632/aging.203294 (PMC8351694; doi:10.18632/aging.203294)
Supplement: Supplementary Table 4 [file aging-13-203294-s005.docx]

**Supplementary Table 4. Code for interactive nomogram.**

**Interactive Nomogram**

**STEP1: R package**

library(pROC)

library(rmda)

library(Hmisc)

library(grid)

library(lattice)

library(Formula)

library(ggplot2)

library(rms)

library(survival)

library(regplot)

library(survminer)

library(timeROC)

**STEP2: read matrix**

dat<- "clinicaldata.txt”

dat <- read.table(file=dat, sep='\t',header = T)

e=exprSet

dat=cbind(dat,e)

dd=datadist(dat)

options(datadist="dd")

**STEP3: Construct nomogram model**

fb <- psm(Surv(time,event) ~ AGE+LDH+ALB+CREAT+Cyto.Abn+riskscore, data =dat, dist='lognormal')

med <- Quantile(fb)

surv <- Survival(fb)

nom <- nomogram(fb, fun=function(x) med(lp=x),

         funlabel="Median Survival Time")

plot(nom)

nom <- nomogram(fb, fun=list(function(x) surv(365, x),

function(x) surv(1095, x),function(x) surv(1825, x), function(x) surv(2555, x)),

funlabel=c("1-year Survival Probability",

"3-year Survival Probability",

"5-year Survival Probability","7-year Survival Probability"))

plot(nom, xfrac=.6)

**STEP4: C-index and ROC curve**

rcorrcens(Surv(time,event) ~ predict(fb), data =  dat)

fcox <- cph(Surv(time, event) ~ AGE+LDH+ALB+CREAT+Cyto.Abn+riskscore, x=T,y=T,surv=T, data=dat)

gfit <- roc(event~predict(fcox), data = dat)

plot(gfit,print.auc=TRUE, print.thres=TRUE, main = "ROC CURVE", col= "red",

print.thres.col="red", identity.col="blue", identity.lty=1,identity.lwd=1)

**STEP5: Calibiration curve**

# Calibiliration curve for f3, f5, f7 are the same code of f1.

f1 <- cph(Surv(time, event) ~ AGE+LDH+CREAT+Cyto.Abn+ALB+riskscore

, x=T, y=T, surv=T, data=dat, time.inc=365)

cal1<- calibrate(f1, cmethod="KM", method="boot", u=365, m=100, B=554)

plot(cal1,lwd=2,lty=4,

errbar.col=c(rgb(0,0,255,maxColorValue=255)),

xlim=c(0.1,0.99),ylim=c(0.1,0.99),

xlab="Nomogram-Predicted Probability of 1-Year OS”

ylab="Actual 1-Year OS (proportion)",

col=c(rgb(255,0,0,maxColorValue=255)))

**STEP6: Interactive nomogram**

fcox <- cph(Surv(time, event==1) ~ AGE+LDH+ALB+CREAT+Cyto.Abn+riskscore, x=T,y=T,surv=T, data=dat)

regplot(fcox,observation=dat[543,], failtime = c(365,730,1095,1258,1825,2555), prfail = T, droplines=F, points=TRUE)
